# No evidence of myocardial restoration following transplantation of mononuclear bone marrow cells in coronary bypass grafting surgery patients based upon cardiac SPECT and ^18^F-PET

**DOI:** 10.1186/1471-2342-6-7

**Published:** 2006-07-14

**Authors:** Paschalis Tossios, Jochen Müller-Ehmsen, Matthias Schmidt, Christof Scheid, Nermin Ünal, Detlef Moka, Robert HG Schwinger, Uwe Mehlhorn

**Affiliations:** 1 Department of Cardiothoracic Surgery, Berufsgenossenschaftliche Kliniken Bergmannsheil, Ruhr-University Bochum, Buerkle-de-la-Camp-Platz 1, 44789 Bochum, Germany; 2 Department of Cardiothoracic Surgery, University of Cologne, Kerpener Str. 62, 50924 Cologne, Germany; 3 Department of Cardiology, University of Cologne, Cologne, Germany; 4 Department of Nuclear Medicine, University of Cologne, Cologne, Germany; 5 Department of Hematology, University of Cologne, Cologne, Germany

## Abstract

**Background:**

We tested the hypothesis, that intramyocardial injection of mononuclear bone marrow cells combined with coronary artery bypass grafting (CABG) surgery improves tissue viability or function in infarct regions with non-viable myocardium as assessed by nuclear imaging techniques.

**Methods:**

Thus far, 7 patients (60 ± 10 [SD] years) undergoing elective CABG surgery after a myocardial infarction were included in this study. Prior to sternotomy, bone marrow was harvested by sternal puncture. Mononuclear bone marrow cells were isolated by gradient centrifugation and resuspended in 2 ml volume of Hank's buffered salt solution. At the end of CABG surgery 10 injections of 0.2 ml each were applied to the core area and borderzones of the infarct. Global and regional perfusion and viability were evaluated by ECG-gated ^99m^Tc-tetrofosmin myocardial single-photon emission computed tomograph (SPECT) imaging and ^18^F-fluorodeoxyglucose positron emission tomography (FDG-PET) in all study patients < 6 days before and 3 months after the intervention.

**Results:**

Non-viable segments indicating transmural defects were identified in 5 patients. Two patients were found to have non-transmural defects before surgery. Concomitant surgical revascularisation and bone marrow cell injection was performed in all patients without major complications. The median total injected mononuclear cell number was 7.0 × 10^7 ^(range: 0.8–20.4). At 3 months ^99m^Tc-tetrofosmin SPECT and ^18^F-FDG-PET scanning showed in 5 patients (transmural defect n = 4; non-transmural defect n = 1) no change in myocardial viability and in two patients (transmural defect n = 1, non-transmural defect n = 1) enhanced myocardial viability by 75%. Overall, global and regional LV ejection fraction was not significantly increased after surgery compared with the preoperative value.

**Conclusion:**

In CABG surgery patients with non-viable segments the concurrent use of intramyocardial cell transfer did not show any clear improvement in tissue viability or function by means of non-invasive bioimaging techniques.

## Background

Patients with coronary artery disease and left ventricular (LV) dysfunction presenting with symptoms of either angina pectoris or heart failure may be considered for coronary artery bypass grafting (CABG) surgery, however, latter is associated with increased risk in this patient population [1]. In a subset of patients with dysfunctional but viable myocardium, both hibernating and stunned, surgical revascularisation has been shown to improve LV function, heart failure symptoms, and survival [2,3]. On the other hand, non-viable myocardium will usually not resume viability and contractile function after standard revascularisation strategies [4-6]. Histopathological verification of viability in patients is impossible. Thus, the ideal methodology to assess myocardial viability would provide accurate non-invasive measurements of perfusion, metabolism, and cellular membrane integrity in addition to LV function [4-8]. Nuclear scintigraphy with single-photon emission computed tomograph (SPECT) imaging technique is used with various tracers to assess perfusion and cell integrity as hallmarks of viability. Most frequently, technetium-99m labelled tracers are injected under resting conditions. In these studies, dysfunctional segments with tracer uptake > 50% are considered viable [8]. The best-validated positron emission tomography (FDG-PET) method for detecting viability assesses both regional metabolism and perfusion. Metabolism is measured by uptake of ^18^F-fluorodeoxyglucose, which is a glucose analogue. It is transported into the cell and is subsequently converted into a compound that is trapped within the myocardium. Normal myocardium is characterized by normal flow, normal glucose uptake, and preferential metabolism of fatty acids over glucose. Infarcted, non-viable myocardium has both decreased flow and glucose uptake [5-7].

The concepts of viability and functional recovery have been used in many patients after myocardial infarction and after standard CABG. However, this issue has not yet been studied in humans in whom stem cell transplantation was performed as an adjunct to cardiac surgery. Hence, detecting resumed viability by using nuclear "bioimaging" techniques in a formerly non-viable myocardial region is of clear scientific and clinical significance and may explain the efficacy of this novel form of cellular therapy that has been developed for myocardial repair in the last decade. These approaches include stem cell therapy with various types of stem cells and delivery mechanisms, in diverse infarction models in animals and humans [9]. In several animal studies, bone marrow cell transplantation has induced angiogenesis, prevented ventricular dilatation, and improved function [10-12]. Clinical studies which evaluate the use of autologous bone marrow cells in conjunction with CABG surgery have generally involved small numbers of patients, and provided preliminary data on the safety and feasibility of targeted implantation, and indicating improved global LV function and myocardial perfusion [13,14]. However, so far, little is known when autologous bone marrow cells are targeted to the core area and borderzones of the myocardial lesion. In this regard, modern non-invasive bioimaging techniques are of paramount importance to evaluate the true extent of myocardial restoration and justify the concurrent use of stem cells and CABG in the same session.

Therefore, the purpose of the present study was to investigate the effects of autologous mononuclear bone marrow cell transplantation following intraoperative bone marrow harvest from the sternum on myocardial viability and function using as widely accepted reference standards ECG-gated ^99m^Tc-tetrofosmin SPECT imaging and ^18^F-FDG-PET in CABG surgery patients.

## Methods

### Patients

Following approval by the University of Cologne Human Ethics Committee (registry #02-025), written, informed consent was obtained from each patient during the preoperative interview. Seven patients with an akinetic infarct region after previous myocardial infarction (>2 weeks) scheduled for elective CABG surgery were included in this study. Patients with unstable angina, valvular disease, or previous CABG were excluded. Before surgery, all study patients underwent ^99m^Tc-tetrofosmin SPECT imaging and ^18^F-FDG-PET at rest for identification of the presence and extent of tissue viability in the infarct region. Three months after CABG surgery and cell transplantation ^99m^Tc-tetrofosmin SPECT and ^18^F-FDG-PET were repeated to assess the degree of restoration of perfusion and myocardial recovery in terms of infarct size reduction.

### ECG-gated ^99m^Tc-tetrofosmin SPECT

For determination of myocardial viability and LV function ECG-gated ^99m^Tc-tetrofosmin (Myoview^®^, Amersham Buchler GmbH & Co.KG, Braunschweig, Germany) SPECT images at rest after oral nitrate medication (Isoket 5^®^, Schwarz Pharma, Monheim, Germany) were acquired. Six hundred MBq ^99m^Tc-tetrofosmin were injected 10 min after nitrate administration. SPECT was performed with a triple-head gamma camera (Prism 3000, Philips Medical Systems, Best, The Netherlands) using a low-energy high resolution collimator in step-and-shoot-modus. For the 360 degree arc 40 projections each with 3 degree difference were acquired with a 64 by 64 matrix and an acquisition time of 30 beats per step. 16 frames were acquired per cardiac cycle. Transaxial images were reconstructed with a slice thickness of 4.12 mm using filtered backprojection (low pass filter, 4^th ^order, cutoff frequency 0.30 cycles/pixel). From the transaxial images short axis, horizontal and vertical long axis slices were reconstructed and a polar map was generated. Attenuation and scatter correction was not performed. LV function was calculated by use of the QGS program (Quantitative gated SPECT, Cedars-Sinai Medical Center, Los Angeles, U.S.A.).

### ^18^F-FDG-PET

^18^F-FDG-PET was performed using a whole-body scanner (Siemens CTI ECAT EXACT 921, Erlangen, Germany). To improve myocardial ^18^F-FDG-uptake, each patient received a solution of 60 g glucose 1 hour before the administration of ^18^F-FDG. Prior to injection of ^18^F-FDG blood glucose levels were checked and a short acting insuline was given according to blood glucose level. Emission scans over 30 minutes were started 45 minutes after injection of 370 MBq F-18-FDG. PET data were corrected for attenuation by using coefficients measured by a transmission scan of 10 minutes duration. PET acquisition data were reconstructed into transaxial tomograms by filtered back-projection employing a Hann filter. Transaxial tomograms were reorientated into cardiac short axis, horizontal and vertical long axis slices and converted into a polar map encompassing the entire left ventricle. Myocardial segments were defined viable by ^18^F-FDG-PET if the mean segmental ^18^F-FDG-uptake was ≥50% compared to a normal reference segment.

### Image analysis

Image analysis was performed using visual and semi-quantitative analysis by polar-tomograms. Relative regional ^99m^Tc-tetrofosmin and ^18^F-FDG-uptake were calculated as percent uptake of the region of interest with maximal uptake (normalisation to the 100% perfusion maximum). To facilitate analysis and to allow clinically relevant conclusions, widely accepted threshold values were applied to distinguish between viable and non-viable myocardium. Concerning ^99m^Tc-tetrofosmin SPECT data, segments were graded as viable if tracer uptake was above a 50% threshhold for anterior infarcts and a 35% threshhold for inferior infarcts and as non-viable if tracer uptake was below 50% or 35% respectively [15]. On ^18^F-FDG-PET, segments were graded as viable if tracer uptake was more than 70% and as non-viable if tracer uptake was below 50%, segments between 50 and 70% were graded as non-transmural defects [16,17]. Both methods (^99m^Tc-tetrofosmin SPECT and ^18^F-FDG-PET) were separately evaluated concerning viability. This was feasible by the concordance of imaging results. An increase in tracer uptake ≥10% after intervention was considered significant and to represent improvement (expressed as percent of the total original defect size). Thereby, the original defect size was considered 100%. Thus, changes in viability reflect both, the ^99m^Tc-tetrofosmin SPECT and ^18^F-FDG-PET results.

### Isolation and cell preparation

**Figure 1:**
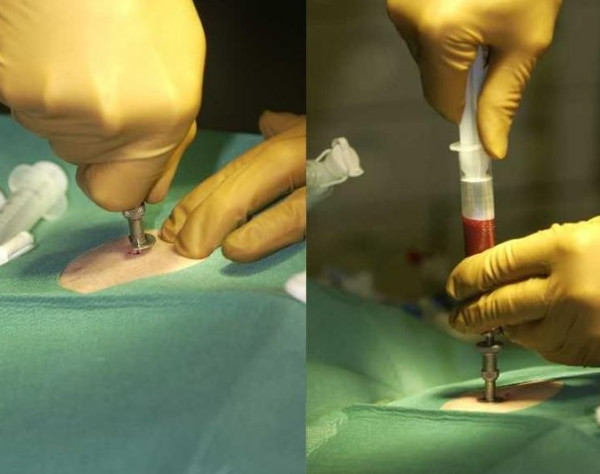
Bone marrow was harvested by sternal puncture prior to sternotomy.

Under general anesthesia and prior to sternotomy, 10 ml of bone marrow was harvested by sternal puncture and 5000 IU Heparin was added (Fig. 1). The cell suspension was density separated by Ficoll and the mononuclear cell fraction was washed twice in Hank's buffered salt solution (HBSS) and resuspended in HBSS to a final volume of 3 ml. While 1 ml was used for flowcytometric characterisation and microbiological testing, 2 ml were returned to the operating room for intramyocardial injection.

### Operative technique and cell transplantation

**Figure 2:**
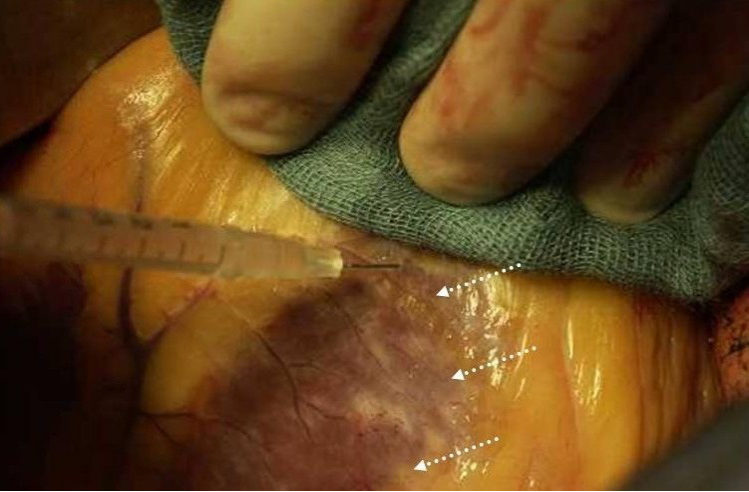
Mononuclear bone marrow cells were applied to the borderzone and the core area of the infarction (arrows).

Patients underwent CABG using standard operative techniques. Myocardial preservation was performed by means of systemic normothermia and warm blood cardioplegia into the ascending aorta before each graft. After completion of the proximal anastomoses, we mobilized the beating and unloaded heart on CPB and injected the suspension of mononuclear bone marrow cells via 10 injections of 0.2 ml each into the infarct region using a standard insulin syringe with a 20-gauge needle (Fig. 2). Nine injections were applied to the periphery of the infarct zone and one injection was applied to the center of the infarct. The targeted injections to infarcted region corresponded to the bioimaging findings which permitted in most cases intraoperative visualization of myocardial scar. We tried to inject the cells allways into the mid-myocardium. The median total injected mononuclear cell number per patient was 7.0 ± 6.4 × 10^7 ^(range: 0.8–20.4). The median percentage of CD34+ progenitor cells was 1.95 ± 0.82% and CD34+/CD133+ 0.8 ± 0.2%, while CD34+/KDR+ endothelial progenitor cells were found with a frequency of 0.02 ± 0.006%.

### Statistical analysis

All data presented are mean ± standard deviation (SD).

## Results

### Patients

Seven patients (5 men and 2 women; mean age of 60 ± 10 years [range: 49 to 76 years]) with angiographically proven coronary artery disease scheduled for CABG surgery were enrolled in this study. All patients had a history of myocardial infarction, and 3 patients had a history of congestive heart failure requiring hospitalisation. The site of myocardial infarction was the posterior wall in 5 patients and the anterior wall in 2. On coronary angiography, 6 patients had three-vessel disease, and 1 patient had two-vessel disease with stenoses of the major coronary arteries of more than 75%. Every patient had a distinct akinetic infarct region determined by left ventriculography or echocardiography, 3 weeks to 8 months after a previous infarction. The LV ejection fraction was 41 ± 7% (range: 31 to 51%). The interval between baseline nuclear imaging and subsequent revascularisation was < 6 days. Clinical and imaging data are shown in Table 1.

### Surgery

An average of 3 ± 1 arteries were grafted (range from 2 to 4). CABG was performed in all patients using the left internal thoracic artery, that was anastomosed to left anterior descending (LAD) (n = 6) and to left circumflex coronary arteries (n = 1). Radial arterial grafts were inserted to left circumflex coronary arteries (n = 3). Venous grafts were used to right coronary arteries (n = 4) and to left circumflex arteries (n = 4) and to diagonal branches (n = 4). All patients were successfully separated from cardiopulmonary bypass. No intraaortic balloon counterpulsation became necessary. No perioperative myocardial infarction occurred.

### Clinical outcomes

Periprocedurally, no major complications occurred in any of our patients. All patients remained free of angina at 3 months follow-up. There were no deaths or subsequent need for revascularisation. One patient was found to have a LV thrombus which had resolved after anticoagulation therapy at the next follow-up. Holter electrocardiograms showed no ventricular arrhythmia up to 3 months after treatment. In addition, echocardiography did not show calcification after intramyocardial cell transplantation.

### Changes in myocardial viability and function

**Figure 3:**
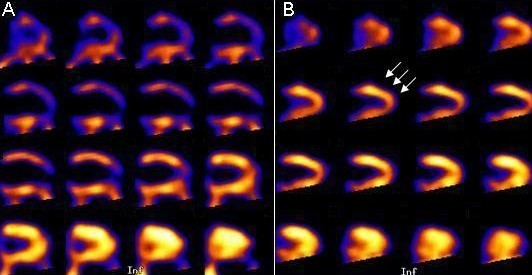
Vertical long axis ^99m^Tc-tetrofosmin SPECT images of patient with myocardial infarction. (A) Perfusion defect at apex and anterior wall before surgical therapy. (B) Three months after cell transplantation and CABG, the apex and anterior wall (arrows) were considered to have normal perfusion.

**Figure 4:**
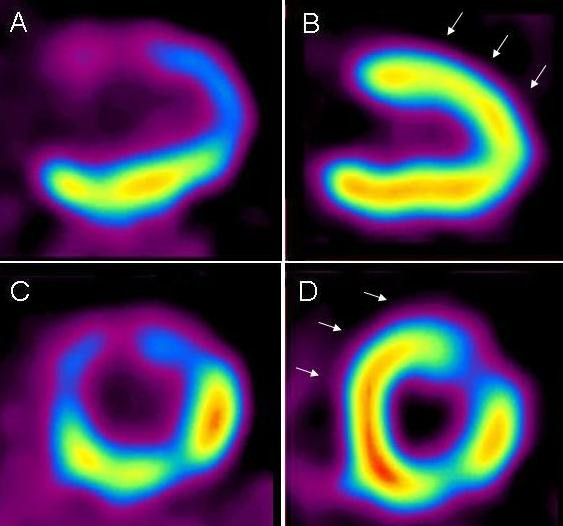
Vertical long axis (A, B) and short axis (C, D) ^18^F-FDG-PET images of patient with myocardial infarction. Non-transmural defect at apex and anterior wall before surgical therapy (A, C). Three months after cell transplantation and CABG, anterior wall and apex showed increased viability (arrows) in infracted area (B, D). EF changed from 31% to 60%.

**Figure 5:**
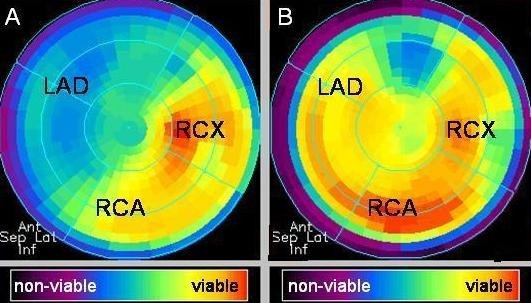
Corresponding polar map obtained from ^18^F-FDG-PET images of patient with myocardial infarction (see Fig. 4). Non-transmural defect at apex and anterior wall before surgical therapy (A). Increased viability in the region of the LAD 3 months after cell transplantation and CABG (B). LAD: left anterior descending coronary artery; RCA: right coronary artery; RCX: circumflex artery.

The relationship between myocardial infarction and the amount of viable myocardium is presented in Table 1. Non-viable segments indicating transmural defects by ^99m^Tc-tetrofosmin SPECT and ^18^F-FDG-PET scans were identified in 5 patients. Two patients were found to have non-transmural defects before surgery. As expected, the patients showed concordant perfusion defects before revascularisation. At 3 months ^99m^Tc-tetrofosmin SPECT and ^18^F-FDG-PET scanning showed in 5 patients (transmural defect n = 4; non-transmural defect n = 1) no change in myocardial viability. Nuclear bioimaging showed in two patients (transmural defect n = 1, non-transmural defect n = 1) enhanced myocardial viability by 75%. Only in the latter patient with a non-transmural defect (Figs. 3, 4, 5) LV ejection fraction improved (31% vs. 60%) after combined CABG and cell therapy. However, overall global and regional LV ejection fraction was not significantly increased at 3 months after surgery compared with the preoperative value (Tabl. 2).

## Discussion

The main findings of our study question the efficacy of autologous cell transplantation for myocardial restoration in CABG surgery patients. This was proven by means of non-invasive bioimaging techniques which showed no evidence of clear improvement in tissue viability or function within akinetic and non-viable infarct regions.

The differentiation between viable myocardium and scar tissue has important clinical implications, because in patients with viable myocardium, revascularisation is likely to result in improvement of regional and global LV function, heart failure symptoms, and long-term prognosis [2,3]. In contrast, regional LV dysfunction arising from transmural myocardial infarction with non-viable myocardium may be irreversible after revascularisation [4-6]. To treat this portion of the ischemic heart muscle a novel therapeutic strategy using bone marrow cells has been proposed as a potential alternative treatment for such patients, because these cells have been shown to improve structure and function in experimental myocardial infarction [11,12]. Thereby, locally released paracrine factors may act by promoting angiogenesis [10].

Preliminary data in small patient numbers in whom bone marrow-derived cells have been injected into myocardial tissue suggest the safety and feasibility of cell transplantation. Hamano et al. [13] injected autologous mononuclear bone marrow cells directly into ungraftable myocardial territories during CABG surgery in 5 patients. Postoperative evaluation revealed no short-term toxicity or adverse events and the engrafted cells seemed to contribute to improved perfusion observed in these territories. In a similar study by Stamm et al. [14], injection of AC 133+ bone marrow cells in the peri-infarct zone in conjunction with CABG in 12 patients resulted in significantly improved global LV function as well as improved perfusion of the infarcted myocardium assessed by thallium scans. Tse et al. [18] found improvement in myocardial perfusion and segmental contractility as assessed by cardiac magnetic resonance imaging in ischemic myocardial segments of 8 patients treated with sole catheter-based intramyocardial implantation of autologous mononuclear bone marrow cells. Silva et al. [19] used transendocardial delivery of autologous bone marrow-derived mononuclear cells in heart transplant candidates with viable ischemic myocardium. After cell therapy exercise capacity improved, and 4 of the 5 patients were no longer eligible for cardiac transplantation. Most recently published studies [20] reported short-term functional improvement of transplantation of mononuclear bone marrow cells through an intracoronary delivery system in patients after successful percutaneous coronary intervention for acute ST-segment elevation myocardial infarct. In most of the studies the ischemic regions injected with cells were still viable and thus, despite the presence of clinically promising data, no conclusion can be drawn for the revitalising capacity of bone marrow cells.

In contrast, in our study injections were performed to non-viable myocardium. We did not find evidence that mononuclear bone marrow cells in combination with CABG are capable of restoring viability or function in patients with formerly non-viable infarcted myocardium. Transepicardial cell injection during open heart surgery into the core and border zone of infarct scar resulted in enhanced myocardial viability by 75% as determined by ^99m^Tc-tetrofosmin SPECT and ^18^F-FDG-PET only in one patient. Though this finding is notable in that metabolically non-viable segments (^99m^Tc-tetrofosmin SPECT and ^18^F-FDG uptake ≤50%) usually do not improve after revascularisation [4-6], the contribution of transplanted mononuclear bone marrow cells to resumed viability is unclear. In this study the median total injected mononuclear cell number as well as the median percentage and the range of CD34+, CD34+/CD133+ and CD34+/KDR+ cells were found to be in concordance with published data from other groups. Interestingly, the number of injected cells did not correlate with the change in myocardial viability.

We used ^99m^Tc-tetrofosmin SPECT and ^18^F-FDG-PET as widely accepted reference standards for the non-invasive assessment of myocardial ischemia and viability [16,17]. Different characteristics such as intact perfusion, cell membrane integrity, and preserved glucose metabolism form the basis for tissue characterisation by these different nuclear bioimaging modalities, that allow to distinguish transmural scar from dysfunctional but viable myocardial tissue [2,4-8]. Thus, scintigraphic viability techniques using combined assessment of perfusion and metabolism offer an advantage over perfusion imaging alone, as for example used by Stamm et al. [14]. However, overall we did not detect global LV function improvement at 3 months after cell transplantation and CABG surgery. From the preliminary data available no difference in regional LVEF before and after surgery could be demonstrated. However, differences in regional LVEF might be better evluated with cine magnetic resonance imaging (MRI) [7].

The major limitations of this ongoing study are the small numbers of patients and the absence of a control group as for most studies published so far. Importantly, all patients underwent cell implantation during bypass surgery. Therefore, an effect of improved myocardial blood flow due to surgical revascularisation itself on the viability as assessed by ^99m^Tc-tetrofosmin SPECT and ^18^F-FDG-PET may be possible. Nevertheless, the preliminary findings reported here, focussing on myocardial viability with nuclear imaging techniques may contribute significantly to the field of stem cell therapy for the treatment of postinfarct patients, since the distinction between viable myocardium and irreversibly damaged scar tissue supplementary to clinical and angiographic data is of profound value. However, given the current incomplete knowledge on the mechanisms of transplanted adult stem cells in vivo, potential benefits and disadvantages must be considered in selecting candidate cells and patients for clinical trials. Clearly, there is a need for randomized, double-blind trials involving large numbers of patients before autologous bone marrow stem cells can be considered a clinically relevant option of therapy.

## Conclusion

Our findings demonstrate that intramyocardial injection of autologous mononuclear bone marrow cells during CABG surgery does not contribute to enhanced myocardial viability or function of akinetic and non-viable infarct regions. The intraoperative bone marrow harvest from the sternum is an efficient and safe method to collect cells for myocardial cell transfer. Further clinical studies are needed to assess the validity of this therapeutic strategy in conjunction with currently used therapies.

## Competing interests

The author(s) declare that they have no competing interests.

## Authors' contributions

Authors PT, JME, RHGS and UM designed the study and drafted the paper.

Authors PT and UM performed surgery and cell transplantation.

Author CS created the method for bone marrow harvesting, performed the cell isolation and preparation and helped write this paper.

Authors MS, NU and DM performed the SPECT and PET image and data analysis and helped with the writing of this paper.

All authors read and approved the final manuscript.

## Pre-publication history

The pre-publication history for this paper can be accessed here:



## Figures and Tables

**Table 1:** Clinical and imaging patient data

Pat	Myocardial defect	Bypass grafts to	Injection location	MNC number	CD34+ cell fraction	Changes in viability
1	Transmural	LAD, D, OM, RCA	Posterolateral	4.2 × 10^7^	1.41%	None
2	Non-transmural	LAD, OM1, OM2	Anterior, apex	0.8 × 10^7^	1.42%	75%
3	Transmural	LAD, D, RCA	Posterobasal	20.4 × 10^7^	1.42%	75%
4	Transmural	LAD, D, OM1	Posterobasal	7.0 × 10^7^	1.26%	None
5	Transmural	LAD, D, OM, RCA	Apex	2.8 × 10^7^	3.28%	None
6	Transmural	OM1, OM2	Posterolateral	7.6 × 10^7^	1.94%	None
7	Non-transmural	LAD, OM, RCA	Posterior	6.0 × 10^7^	2.93%	None

D: diagonal coronary artery; LAD: left anterior descending coronary artery; OM: obtuse marginal coronary artery; MNC: mononuclear cell; Pat: patient; RCA: right coronary artery.

**Table 2:** LV volumes measured by gated SPECT

Pat	EDV pre.	EDV post.	ESV pre.	ESV post.	SV pre.	SV post.	EF pre.	EF post.
1	136 ml	172 ml	88 ml	115 ml	47 ml	57 ml	35%	33%
2	174 ml	101 ml	120 ml	41 ml	54 ml	60 ml	31%	60%
3	134 ml	116 ml	69 ml	57 ml	65 ml	59 ml	48%	51%
4	108 ml	124 ml	66 ml	72 ml	42 ml	52 ml	39%	42%
5	136 ml	209 ml	88 ml	134 ml	47 ml	75 ml	35%	36%
6	n.a.	115 ml	n.a.	58 ml	n.a.	57 ml	46%*	50%
7	99 ml	128 ml	49 ml	74 ml	50 ml	54 ml	51%	42%

EDV: end diastolic volume; EF: ejection fraction; ESV: end systolic volume; LV: left ventricular; n.a.: not available; Pat: patient; post: postoperative; pre: preoperative; SPECT: single-photon emission computed tomography; SV: stroke volume; *: EF was assessed by echocardiography.
